# Effects of Kimchi Intake on the Gut Microbiota and Metabolite Profiles of High-Fat-Induced Obese Rats

**DOI:** 10.3390/nu16183095

**Published:** 2024-09-13

**Authors:** Dong-Wook Kim, Quynh-An Nguyen, Saoraya Chanmuang, Sang-Bong Lee, Bo-Min Kim, Hyeon-Jeong Lee, Gwang-Ju Jang, Hyun-Jin Kim

**Affiliations:** 1 Division of Applied Life Sciences (BK21 Plus), Gyeongsang National University, Jinju 52828, Republic of Korea; kimdw5791@korea.kr (D.-W.K.); an.ngqnh@gmail.com (Q.-A.N.); sblee@kfri.re.kr (S.-B.L.); qhals159@naver.com (B.-M.K.); hjl@kist.re.kr (H.-J.L.); gjjang@kfri.re.kr (G.-J.J.); 2 Food Safety and Processing Research Division, National Institute of Fisheries Science, Busan 46083, Republic of Korea; 3 Department of Food Science and Technology, Institute of Agriculture and Life Science, Gyeongsang National University, 501 Jinjudaero, Jinju 52828, Republic of Korea; c.saoraya@gmail.com; 4 Food Safety and Distribution Research Group, Korea Food Research Institute, Wanju-gun 55365, Republic of Korea; 5 Doping Control Center, Korea Institute of Science and Technology, Seongbuk-gu, Seoul 02792, Republic of Korea

**Keywords:** gut microbiota, kimchi, metabolomics, obesity, steroid hormones

## Abstract

With rising global obesity rates, the demand for effective dietary strategies for obesity management has intensified. This study evaluated the potential of kimchi with various probiotics and bioactive compounds as a dietary intervention for high-fat diet (HFD)-induced obesity in rats. Through a comprehensive analysis incorporating global and targeted metabolomics, gut microbiota profiling, and biochemical markers, we investigated the effects of the 12-week kimchi intake on HFD-induced obesity. Kimchi intake modestly mitigated HFD-induced weight gain and remarkably altered gut microbiota composition, steroid hormones, bile acids, and metabolic profiles, but did not reduce adipose tissue accumulation. It also caused significant shifts in metabolomic pathways, including steroid hormone metabolism, and we found substantial interactions between dietary interventions and gut microbiota composition. Although more research is required to fully understand the anti-obesity effects of kimchi, our findings support the beneficial role of kimchi in managing obesity and related metabolic disorders.

## 1. Introduction

Kimchi, a traditional fermented food made from salted vegetables, such as cabbage and radish, and flavored with various seasonings, is celebrated for its health benefits. It is a rich source of vitamins, minerals, dietary fibers, phytochemicals, and probiotics, including diverse species of lactic acid bacteria (LAB), such as *Leuconostoc*, *Lactobacillus*, and *Weissella* species [1]. Many clinical and animal studies have linked the kimchi intake with the prevention or mitigation of several diseases, including hypercholesterolemia, cancer, inflammation, diabetes, and obesity [2]. In particular, recent randomized controlled clinical trials in overweight and obese patients have revealed a positive association between the intake of fresh or fermented kimchi and weight maintenance as well as a reduction in obesity-related disorders such as increased blood glucose, blood pressure, insulin, and total cholesterol levels [3]. Furthermore, kimchi intake over two four-week diet phases separated by a two-week washout period resulted in weight loss and improved metabolic parameters in overweight and obese patients [4]. However, in a kimchi intervention study, none of the clinical parameters associated with obesity, including body weight, body fat, triglyceride levels, and blood pressure, significantly changed after eight weeks of fermented kimchi intake [3], while changes in gut microbiota profiles and gene expression levels were observed. Although kimchi dietary fiber, capsaicin, and LABs have been recognized as potential anti-obesity agents [5,6], scientific evidence supporting this relationship is scarce, and the underlying mechanism remains unclear.

Recent advances in multi-omics technologies have enabled a deeper understanding of the complex biochemical and molecular changes associated with obesity and obesity-related disorders [7,8]. In our previous studies, we analyzed the metabolite profiles of obese patients and high-fat diet (HFD)-induced obese mouse models and suggested an obesity-related pathway, including energy metabolism through fatty acid oxidation [9]. Moreover, accumulating evidence suggests that the gut microbiota, which affects metabolism and energy balance, may contribute to obesity and metabolic disorders in the host [10]. However, the effects of kimchi intake on metabolites and gut microbiota in an obese model has not been investigated, except for an intervention study involving fresh and fermented kimchi, which examined the gut microbiota composition and gene expression in obese women [3].

Therefore, in this study, the global and targeted metabolites and gut microbiota profiles of HFD-induced mildly obese rats fed with fermented kimchi were analyzed to better understand the correlation between kimchi intake and obesity. Moreover, metabolomic pathways, including the steroid hormone pathway, were proposed based on metabolite analysis.

## 2. Materials and Methods

### 2.1. Kimchi

Kimchi was obtained from Gamchilbaegi Corp. (Gwangju, Republic of Korea). Briefly, Napa cabbage (*Brassica* rapa ssp. *pekinensis*) was brined for 12 h and subsequently drained for 12 h. It was then mixed with other ingredients (radish, red pepper, leaf mustard, onions, garlic, ginger, pears, green onion, fermented fish sauce, kelp extract, sticky rice soup, sugar, and sea salt) and fermented at 10 °C for five days. After fermentation, the kimchi (pH, 4.9; acidity, 0.6%; bacterial count, 8.6 log CFU/g) was lyophilized and stored at −80 °C until analysis.

### 2.2. Animal Study

This animal study was approved by the Animal Research Committee of Gyeongsang National University (GNU-151012-R0061). Four-week-old male Sprague Dawley rats were purchased from Koateck (Pyeongtaek, Republic of Korea). After two weeks of adaptation, the rats were randomly divided into three groups, and each group (*n* = 10) was fed one of the following three diets for 12 weeks: normal diet (ND; 2018S Teklad Global 18% Protein Rodent Diet, Envigo, IN, USA), HFD (D12492, 60% kcal; Research Diets, New Brunswick, NJ, USA), and HFD with 5% kimchi powder (KHD). Throughout the experimental period, the rats were kept in a temperature-regulated room with a 12 h light and dark cycle. The rats had unrestricted access to food and water. The food intake was monitored daily, and body weight was measured weekly. Urine was collected over a 12 h period in a metabolic cage containing 0.1% sodium azide. After the rats were anesthetized using diethyl ether and blood was collected using a vacuette blood collection tube from the postcaval vein, the rats were euthanized through an overdose of the anesthetic. The large intestinal residues and feces were collected and immediately placed in liquid nitrogen. The kidney, liver, and epididymal adipose tissues were harvested, rinsed with phosphate-buffered saline, wiped with paper towels, weighed, and promptly placed in liquid nitrogen. Liver, kidney, and urine samples were freeze-dried. All samples were stored at −70 °C until analysis.

### 2.3. Biochemical Markers

Plasma triglyceride (TG), total cholesterol (TC), low-density lipoprotein (LDL), high-density lipoprotein (HDL), aspartate aminotransferase (AST), and alanine aminotransferase (ALT) levels were measured using enzymatic assay kits (Asan Pharmaceutical Co., Seoul, Republic of Korea). After 11 weeks, an oral glucose tolerance test was conducted on 12 h fasted rats. Blood glucose levels were measured at 0, 30, 60, 90, and 120 min after oral administration of a glucose solution (2 g/kg body weight) using an Accu-Check glucometer (Roche, Mannheim, Germany) on blood drawn from the tail vein.

### 2.4. Analysis of Gut Microbiota

The gut microbiota in rats were analyzed according to a previously described method [11] with minor modifications. Briefly, bacterial DNA was isolated from rat feces using a QIAamp DNA Stool Mini Kit (Qiagen, Hilden, Germany). The V1–V2 region of the 16S rDNA genes was amplified by polymerase chain reaction (PCR) using a C1000 thermal cycler (Bio-Rad, Hercules, CA, USA), Maxime PCR PreMix (INtRON Biotechnology, Seongnam-Si, Republic of Korea) DNA libraries were constructed using the Ion Xpress Plus Fragment Library Kit (Thermo Scientific, Wilmington, DE, USA), and their concentrations were quantified using a Bioanalyzer 2100 instrument (Agilent Technologies, Inc., Santa Clara, CA, USA) with high-sensitivity DNA chips. Sequencing was performed using the Ion Torrent PGM system (Thermo Scientific). Quantitative Insights Into Microbial Ecology (QIIME2, version 2020.08, Flagstaff, AZ, USA) as used to process and analyze the sequences, and amplicon sequence variants were assigned using the SILVA v138 database [12,13]. To assess alpha diversity, indices such as Chao1 and Shannon were calculated, while beta diversity was assessed through weighted UniFrac distances using the QIIME beta-diversity script (QIIME, Flagstaff, AZ, USA).

### 2.5. Global Metabolomic Analysis

Mass-based metabolomic analysis was carried out according to a previously described method [11] with minor modifications. For liquid chromatography–mass spectrometry (LC–MS), plasma samples were mixed with cold acetone (1:1, *v*/*v*) to remove proteins, and the supernatant was fully dried. Dried samples were dissolved in 20% methanol containing terfenadine as an internal standard (IS). Similarly, lyophilized urine was dissolved in distilled water, and lyophilized kidney, liver, and large intestinal residues were homogenized in 70% methanol with the IS. After centrifugation, the supernatants were dried, and then the residues were dissolved in 20% methanol. All supernatants were analyzed using ultra-performance liquid chromatography-quadrupole time-of-flight (UPLC-Q-TOF) MS (Waters, Milford, MA, USA) with a Acquity UPLC BEH C18 column (2.1 mm × 100 mm, 1.7 um; Waters). The mobile phase consisted of 0.1% formic acid in water (A) and acetonitrile in 0.1% formic acid (B). The eluents ionized by positive electrospray ionization (ESI) were analyzed using a Q-TOF mass spectrometer under the following optimized conditions: capillary voltage of 3 kV, sampling cone voltage of 40 V, desolvation flow rate of 800 L/h at 400 °C, and source temperature of 120 °C.

For gas chromatography (GC)–MS, the lyophilized urine was incubated with 34 units of urease (Sigma Aldrich, St Louis, MO, USA) at 37 °C for 30 min. After urease precipitation with the addition of 50 μL of methanol, the samples were dried and derivatized in 70 μL of 2% methoxyamine hydrochloride in pyridine with dicyclohexyl phthalate as the IS at 37 °C for 90 min, and treated with 70 μL of N,O-bis(trimethylsilyl)trifluoroacetamide (BSTFA) containing 1% trimethylchlorosilane (TMSC) at 70 °C for 30 min. Derivatized samples were analyzed using GC–MS (Shimadzu, Tokyo, Japan) with a DB-5ms capillary column (30 m × 0.25 mm, Agilent J&W, Santa Clara, CA, USA). The oven temperature was ramped up from 80 °C to 320 °C. The effluent was detected using a GCMS-TQ 8030 MS (Shimadzu) in the electron ionization (EI) mode (70 eV) at the source and interface temperatures of 230 °C and 280 °C, respectively.

LC–MS data were aligned based on mass tolerance and retention time, and then normalized using the IS. Metabolites were identified using ChemSpider (www.chemspider.com) and authentic standards. Metabolites were identified by retention indices (RIs) calculated using C8–C40 *n*-alkanes and Wiley and NIST mass spectral databases, along with authentic standards.

### 2.6. Steroid Hormone and Bile Acid Analysis Using UPLC-Q-TOF MS

Steroid hormones and bile acids were analyzed using a previously reported method [11]. To extract steroid hormones from urine and bile acids from feces, the lyophilized urine powder or fecal samples were mixed with 80% methanol containing estradiol-d5 and zidovudine, respectively, as internal standards and sonicated for 30 min. After centrifugation, the supernatants were analyzed using UPLC-Q-TOF MS in the multiple reaction monitoring (MRM) mode (Supplementary Tables S1 and S2). The column and UPLC-Q-TOF MS conditions were the same as those used for the metabolite analysis described above.

### 2.7. Analysis of Short-Chain Fatty Acids (SCFAs) by GC–MS

Fecal short-chain fatty acids (SCFAs) were determined and quantified using GC following a modified version of a previously described protocol [14]. To extract SCFAs, 30 mg of fecal sample was homogenized with 300 µL of hexane and then sonicated for 5 min. After centrifugation, 200 µL of the supernatant was mixed with 100 µL of distilled water, 10 µL of HCl, and 400 µL of ether. After shaking for 5 min at 4 °C, the ether layer was derivatized in a GC vial with 20 µL of BSTFA at 70 °C for 20 min, and then at 37 °C for 2 h. The derivatized samples were analyzed using a GC-2010 Plus system (Shimadzu) equipped with a DB-5ms capillary column (Agilent J&W). The ion source and interface temperatures were 200 °C and 250 °C, respectively. SCFAs were quantitatively analyzed using authentic standards.

### 2.8. Real-Time PCR

To analyze inflammation-related cytokines in the colon, total RNA was extracted from the rat colon tissue using the RNAeasy Mini Kit (Qiagen), and cDNA was synthesized using a High-Capacity cDNA Reverse Transcription Kit (ThermoFisher Scientific) at 25 °C for 10 min, 37 °C for 120 min, and 85 °C for 5 min. Real-time PCR was carried out using the QuantStudio™3 Real-Time PCR System (Applied Biosystems, Foster City, CA, USA) with PowerUp™ SYBR™ Green Master Mix (ThermoFisher Scientific) and the following specific primers: interleukin-12 (*IL-12*); interleukin 1 beta (*IL-1b*); interleukin-6 (*IL-6)*; interferon gamma (*IFN*-*γ*); tumor necrosis factor alpha (*TNF-α*); and actin beta (*Actb*) (Supplementary Table S3). Gene expression was calculated relative to *Actb* [11].

### 2.9. Statistical Analysis

Multivariate statistical analysis of the processed MS data was conducted using SIMCA-P^+^ version 12.0.1 (Umetrics, Umeå, Sweden), and one-way analysis of variance (ANOVA) was performed with Duncan’s test (*p* < 0.05), using SPSS 17.0 (SPSS Inc., Chicago, IL, USA). Moreover, the biological characteristics of the rats, real-time PCR data, and normalized intensities of global and targeted metabolites were analyzed using ANOVA and Duncan’s test (*p* < 0.05). Correlations between gut microbiota and metabolites were analyzed and visualized using GraphPad Prism v8 heat maps.

## 3. Results

### 3.1. Biological Characteristics

The general biological characteristics of rats fed the ND, HFD, and KHD for 12 weeks were investigated (Table 1). The HFD increased the body weight (273.57 g) and epididymal adipose tissue weight (9.30 g) compared to those of the ND (body weight: 199.55 g; adipose tissue weight: 5.63 g). However, rats fed with the HFD and KHD exhibited a reduced food intake of approximately 30 g/day compared to that of the ND (46.81 g/day). The KHD reduced the HFD-induced body weight, and the body weight induced by the KHD (208 g) was similar to that of the ND. Notably, the KHD did not affect the epididymal adipose tissue weight increase induced by the HFD. The blood ALT and TG contents of rats were decreased to 28.88 g and 36.14 g, respectively, by the HFD compared to those by the ND (38.33 g and 45.11 g, respectively), but the KHD did not significantly affect the contents decreased by the HFD. None of the other biological characteristics, including tissue weight, AST, and cholesterol, were significantly altered by the HFD or the KHD. Oral glucose tolerance test data showed that the kimchi diet tended to lower the blood glucose level elevated by the HFD compared to the ND group (Supplementary Table S4). However, no significant difference was observed between the KHD and HFD groups except at 120 min.

### 3.2. Gene Expression Levels of Inflammatory Cytokines in the Colon

The expression levels of genes involved in inflammation, including *IL-12*, *IL-6*, *IL-1b*, *TNFα*, and *IFN*-*γ*, were determined in the colon (Supplementary Figure S1). The expression of *IL-12*, *IL-1b*, and *IFN*-*γ* genes was increased by 3 to 4.7 times by the HFD compared to those of the ND, but no significant decrease in HFD-induced cytokine expression levels was observed after KHD treatment.

### 3.3. Gut Microbiota

**Figure 1:**
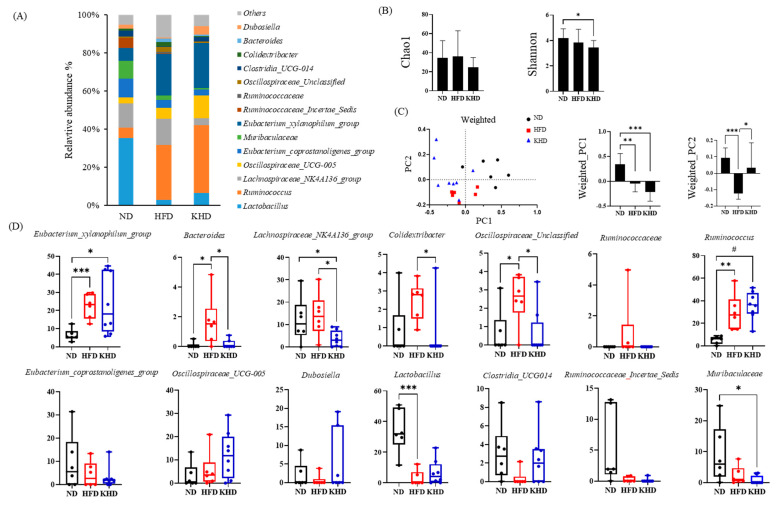
Comparison of gut microbiota’s relative abundance after 12 weeks of kimchi intervention. (**A**) Bar charts showing the overall microbial composition at genus levels in feces from rats fed ND, HFD, and KHD diets, with the average relative abundance. (**B**) Chao1 and Shannon indices calculated after rarefying to an equal number of sequence reads. (**C**) Principal-coordinate analysis plots of weighted UniFrac distance dissimilarities (PC1 and PC2). (**D**) Relative abundances of bacteria at the genus level. ND, control; HFD; high-fat diet; KHD, high-fat-kimchi diet. * *p*-value < 0.05, ** *p*-value < 0.01, *** *p*-value <0.001, # *p*-value < 0.0001. Data represent the relative abundance of microbes analyzed from six samples. Different letters on the bar and box plot indicate significant differences in the *t*-test at *p* < 0.05.

The gut microbiota of the three groups were analyzed, and their profiles and relative abundances were compared (Figure 1 and Supplementary Figure S2). The Shannon index showed that the diversity of the gut microbiota in the KHD group was significantly lower than that in the ND group, whereas the richness evaluated using the Chao1 index was not significantly different among the groups. Notably, the three groups were significantly separated on the weighted plots by PC1 and PC2, and the KHD group showed a significant recovery in the direction of PC2 when compared with the HFD group in the weighted UniFrac. To identify the microbiota contributing to the separation, the relative abundances of individual bacteria were compared at the genus level. The populations of *Eubacterium_xylanophilum_group*, *Bacteroides*, *Lachnospiraceae_NK4A136_group*, *Colidextribacter*, *Oscillospiraceae_Unclassfied*, *Ruminococcaceae*, and *Ruminococcus* increased by 2.1 to 11.8 times in the HFD group compared to those in the ND group, while other microbiota populations decreased. KHD partially restored these populations, except for *Eubacterium_coprostanoligenes_group*, *Ruminococcaceae_Incertae_Sedis*, and *Muribaculaceae*.

### 3.4. Global Metabolites and SCFAs

**Figure 2:**
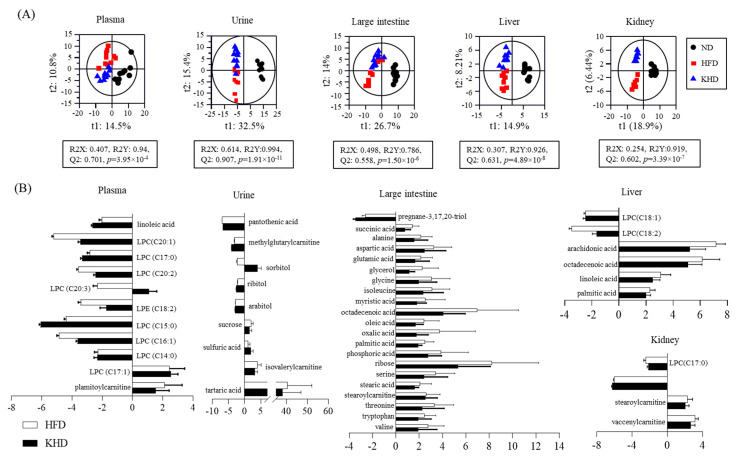
(**A**) Metabolite analysis of rats fed HFD and KHD. Partial least-squares discriminant analysis (PLS-DA) score plot obtained from UPLC-Q-TOF MS data of plasma, urine, large intestine, liver, and kidney (*n* = 10). (**B**) Fold change of identified metabolites. Metabolites were analyzed using UPLC-Q-TOF MS via an Acquity BEH C18 column (2.1 mm × 100 mm, 1.7 μm) with a positive ESI mode. The qualification of PLS-DA models was evaluated by R2X, R2Y, Q2, and *p*-values. R2X and R2Y show the fitting quality of the models, while Q2 shows their prediction quality. ND, control; HFD; high-fat diet; KHD, high-fat-kimchi diet.

**Figure 3:**
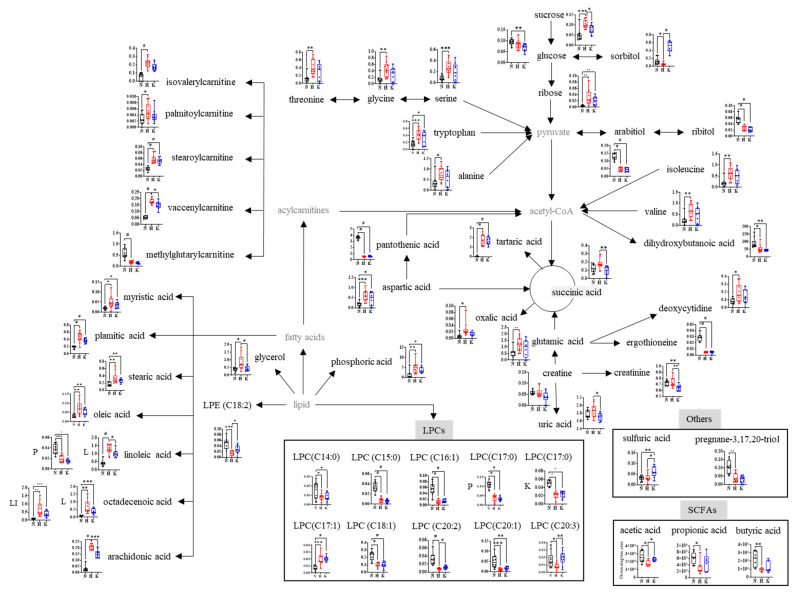
Proposed metabolomic pathway associated with HFD and kimchi intake and the relative abundance of metabolites. Box plots present the relative abundance of metabolites analyzed by UPLC-Q-TOF MS, with significant differences determined by *t*-tests at *p*-values < 0.05 (*), <0.01 (**), <0.001 (***), and <0.0001 (#). N, normal-diet group; H, high-fat-diet group; K, kimchi-high-fat-diet group; P, plasma; L, liver; K, kidney; LI, large intestinal residues.

The metabolite profiles of plasma, urine, liver, kidney, and large intestinal residues from rats fed the ND, HFD, and KHD were analyzed by LC–MS and GC–MS, and their datasets were statistically analyzed using PLS-DA (Figure 2A). The quality parameters (0.786 < R2Y < 0.994, 0.515 < Q2 < 0.907, 1.91 × 10^−11^ < *p*-value < 3.95 × 10^−4^) and cross-validation data (*Y*-axis of R2 < 0.5 and Q2 < −0.3; Supplementary Figure S3) for all samples indicated that all the PLS-DA models were statistically acceptable. The three groups were clearly separated based on the PLS-DA score plots by t1 and t2 values. Among the metabolites detected by LC–MS and GC–MS, 159, including lipids, amino acids, purines, sugar-related metabolites, and acidic compounds, were identified as the major metabolites contributing to the separation of the three groups, and their fold changes versus ND were calculated (Supplementary Tables S5 and S6). Among these metabolites, the levels of 50 metabolites were altered more than two-fold by the HFD and/or KHD compared to the ND (Figure 2B). Based on these metabolites, a metabolomic pathway related to the HFD and kimchi intake was proposed, and the relative abundance of each metabolite was compared (Figure 3). In the plasma, the levels of palmitoylcarnitine and lysophosphatidylcholines (LPCs) (C17:1) increased two-fold in the HFD group compared to those in the ND group, whereas LPCs (C14:0, C15:0, C16:1, C17:0, C20:1, C20:2, and C20:3), LPE C18:2, and linoleic acid levels decreased two- to five-fold. In particular, the fold changes in LPCs (C16:1, C20:1, C20:2, and C20:3) were partially recovered by the KHD. However, other compounds were negatively affected or unaffected by the KHD when compared with the HFD. In urine, the levels of sulfuric acid, isovalerylcarnitine, and tartaric acid were increased 1, 4, and 40.5 times, respectively, by the HFD, whereas pantothenic acid, methylglutaryl carnitine, and polyol (arabitol, ribitol, and sorbitol) levels decreased from 2.1 to 7 times. However, most urinary metabolites were not recovered by the KHD, except for sorbitol, which was decreased by 2.2 times with the HFD but increased by 4 times with the KHD. Moreover, the KHD increased sulfuric acid levels by 1.9 times compared to the ND and HFD. In the liver, the HFD increased the levels of fatty acids (palmitic acid, linoleic acid, octadecenoic acid, and arachidonic acid) by 2.3, 3.1, 6.2, and 7.2 times, respectively, whereas LPE (C18:2) and LPC (18:1) levels decreased by 3.5 and 2.5 times, respectively. The KHD partially restored the levels of all metabolites, except for LPC (18:1). In the kidneys, the HFD increased the levels of stearoylcarnitine and vaccenylcarnitine by 2.3 and 3.1 times, respectively, compared to the ND, whereas ergothioneine and LPC levels (C17:0) decreased by 6.1 and 2.5 times, respectively. However, the renal metabolites affected by the HFD were not restored by the KHD. In the intestinal residues, the HFD increased the levels of amino acids (tryptophan, alanine, valine, isoleucine, serine, threonine, glycine, and aspartic acid), fatty acids (myristic acid, glutamic acid, palmitic acid, oleic acid, stearic acid, and octadecenoic acid), stearoylcarnitine, oxalic acid, phosphoric acid, glycerol, and ribose by two to eight times. These levels increased by the HFD were partially restored by the KHD. However, pregnane-3,17,20-triol levels were decreased by the HFD and KHD by 2.6 times and 3.4, respectively. Moreover, compared to the ND, the HFD remarkably decreased the levels of SCFAs, including butyric acid, propionic acid, and acetic acid, by 1.5, 2.4, and 1.9 times, respectively (Figure 3). However, the SCFA levels reduced by the HFD were increased by the KHD.

### 3.5. Steroid Hormone and Bile Acid Profile

**Figure 4:**
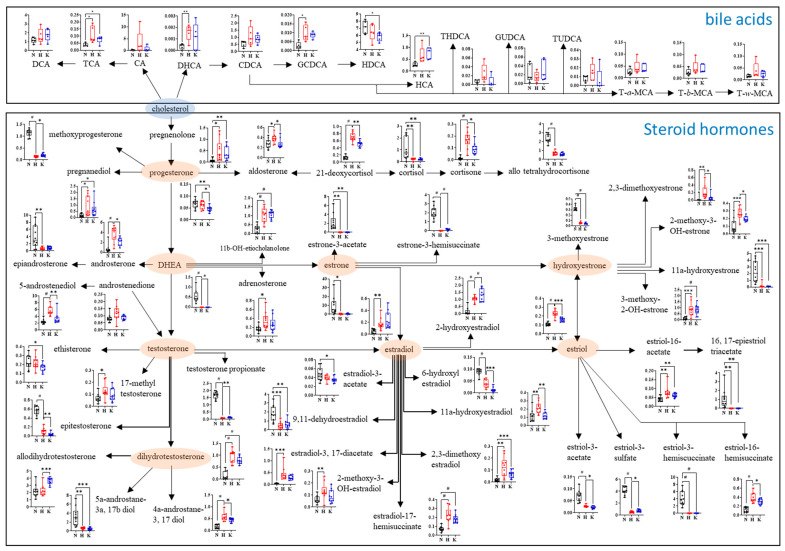
Proposed bile acid and steroid hormone pathway associated with HFD and kimchi intake and their relative abundances. Box plots present the relative abundance of bile acids and steroid hormones analyzed by UPLC-Q-TOF MS, with significant differences determined using *t*-tests at *p*-values of <0.05 (*), < 0.01 (**), <0.001 (***), and <0.0001 (#). N, normal-diet group; H, high-fat-diet group; K, kimchi-high-fat-diet group.

Forty-seven urinary steroid hormones, which were analyzed using UPLC-Q-TOF MS in the MRM mode, were significantly affected by the HFD or KHD. A steroid hormone pathway was proposed based on these steroid hormones, and their relative abundances were analyzed (Figure 4). The HFD and KHD significantly altered most urinary steroid hormones when compared with the ND. The HFD increased the levels of estriol, androsterone, estriol-16-hemisuccinate, 4a-androstane-3,17b-diol, 21-deoxycortisol, adrenosterone, 2-methoxy-3-OH-estradiol, estradiol-3,17b-diacetate, 2,3-dimethoxyestradiol, 3-methoxy-2-OH-estrone, cortisone, 11b-OH-etiocholanolone, 2,3-dimethoxyestrone, 2-methoxy-3-OH-estrone, 2-methoxy-3-OH-estrone, dihydrotestosterone, pregnenolone, estradiol-17-hemisuccinate, pregnanediol, and 5-androstenediol by more than two times. These levels were partially restored by the KHD, except for estradiol, 2-hydroxyestradiol, 11b-OH-etiocholanolone, and 3-methoxy-2-OH-estrone. In contrast, most other steroid hormones were decreased by the HFD. Notably, the levels of epitestosterone, 6-hydroxyestradiol, DHEA, allo tetrahydrocortisone, 11a-hydroxyestrone, 3-methoxyestrone, estradiol-3,17a-diacetate, estriol-3-hemisuccinate, estrone-3-acetate, and 16,17-epiestriol triacetate were further reduced by the KHD.

In addition to steroid hormones, the HFD increased the levels of most stool bile acids by 1.4 to 12.4 times, except for hyodeoxycholic acid (HDCA) and glycoursodeoxycholic acid (GUDCA), compared to the ND (Figure 4). Among the elevated bile acids, the levels of cholic acid (CA), dehydrocholic acid (DHCA), taurohyodeoxycholic acid (THDCA), and tauroursodeoxycholic acid (TUDCA) tended to decrease following the KHD. However, no significant differences in the levels of other bile acids were observed between the HFD and KHD groups. In contrast, deoxycholic acid (DCA), hyocholic acid (HCA), and GUDCA levels increased in response to the KHD.

### 3.6. Correlations between Gut Microbiota, Bile Acids, Steroid Hormones, and Metabolites

**Figure 5:**
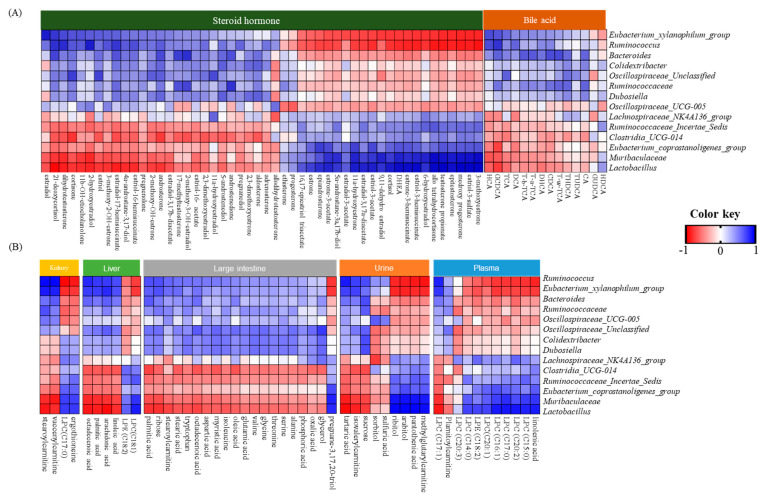
Analysis of correlations of gut microbiota with bile acid and steroid hormones (**A**), and gut microbiota with identified metabolites’ data (**B**). The correlation matrix was analyzed and visualized with a heat map. Positive correlations are shown in blue, and negative correlations are shown in red. A dark color means a stronger correlation.

Correlations among the gut microbiota, bile acids, steroid hormones, and metabolites were analyzed (Figure 5). Most gut microbiota showed either a positive or negative correlation with these factors.

Eubacterium_xylanophilum_group, Ruminococcus, Bacteriodes, Colidextribacter, Oscillospiraceae_Unclassified, Ruminococcaceae, and Dubosiella were negatively correlated with steroid hormones, including 3-methoxyestrone, estriol-3-sulfate, medroxyprogesterone, epitestosterone, testosterone propionate, alloterahydrocortisone, 6-hydroxyestradiol, estriol-3-hemisucinate, estrone-3-hemisucinate, dehydroepiandrosterone (DHEA), cortisol, 9,11-dehydroestradiol, estriol-3,17a-diacetate, 11a-hydrosyestrone, estrone, and 16,17-epiestrol triacetate (r = −0.84 to −0.62), but positively correlated with other isolated steroid hormones and most bile acids, except for HDCA and GUDCA. However, other isolated bacteria showed the opposite correlations with steroid hormones and bile acids.

Additionally, linoleic acid in the blood, methylglutarylcarnitine, pantothenic acid, and polyols in the urine, pregnane-3,17,20-triol in the large intestine, ergothioneine in the kidney, and most lysophospholipids had a positive correlation with *Muribaculaceae*, *Lactobacillus*, *Eubacterium_xylanophilum_group*, *Ruminococcaceae_Incertae_Sedis*, *Lachnospiraceae_NK4A136_group*, and *Clostridia_UCG-014* (r = 0.62 to 0.96). However, other metabolites showed a negative correlation with them (r = −0.90 to −0.56). Other isolated bacteria were correlated with other identified metabolites in the opposite direction.

## 4. Discussion

In this study, our findings revealed that a 12-week HFD led to mild obesity, characterized by increased body weight and adipose tissue compared to the ND, without significantly altering other biochemical markers (Table 1). Notably, kimchi intake only partially mitigated HFD-induced weight gain, while it significantly influenced other parameters, including the gut microbiota composition, steroid hormones, bile acids, and metabolites. The observed anti-obesity effect of kimchi intake was consistent with the results of a kimchi microbial community intervention study [15]. Moreover, the present study uniquely identified parameters that have not been previously reported.

The HFD notably altered the gut microbiota and increased the populations of *Bacteroides*, *Lachnospiraceae*_NK4A136 group, *Colidextribacter*, and *Oscillospiraceae*_Unclassified, while reducing *Clostridia_UCG-014* and *Lactobacillus* levels (Figure 1). Interestingly, these changes were partially reversed in the KHD group. *Lachnospiraceae* have been implicated in the development of obesity and diabetes in mice [16] and are associated with obesity-related metabolic disorders in older adults [17], despite conflicting evidence regarding their role in human gut health [18]. Additionally, *Oscillospiraceae* [19,20], *Bacteroides* [21], and *Colidextribacter* [22] have been linked to obesity and metabolic disorders, and their abundance is inversely correlated with health indicators such as blood pressure, glucose, and triglyceride levels. Conversely, specific *Lactobacillus* strains, including *L. reuteri* L3 and *L. casei Shirota*, have demonstrated anti-obesity effects through body weight, fat storage, and inflammation reduction [23,24,25] and have been linked with metabolic phenotype modulation, lipid metabolism improvement, and adipocytokine regulation. Our findings contrast with data on *Clostridia*, where obesity correlates with an increased infection risk from specific species, such as *Clostridium difficile* [26,27]. The presence of *Clostridium ramosum* has been linked to obesity and metabolic syndrome [28], with the HFD amplifying *Clostridium* cluster XIVa in obese rodent gut microbiota [29]. Similar to previous studies [24,30], these alterations in gut microbiota were positively correlated with elevated inflammatory cytokine levels in the HFD group (Supplementary Figure S1). However, despite the partial restoration of the bacterial profile in the KHD group, cytokine levels were not significantly altered, suggesting that other factors may influence inflammatory responses.

Kimchi intake partially restores body weight and gut microbiota altered by the HFD, potentially due to the LABs and non-digestible carbohydrates (NDCs) in kimchi [15]. Previous studies have demonstrated the potential of kimchi LAB strains, including *Leuconostoc mesenteroides* and *Lactobacillus sakei*, in combating obesity. Supplementation with kimchi LABs reduces obesity-related parameters in mice, including blood urea nitrogen, glucose, and triglyceride levels, as well as fatty liver disease [5], and in individuals with obesity [31]. Similarly, kimchi *Lactobacillus brevis* OPK-3 prevents weight gain and reduces inflammatory cytokine levels in the adipose tissue of mice [30]. Furthermore, many clinical and animal studies have suggested that NDC intake increases intestinal viscosity, fecal bulking, and SCFA production via gut fermentation, resulting in the suppression of obesity-associated health problems (Figure 3). Our study also showed that the HFD led to a decrease in the population of SCFA-producing bacteria, such as *Lactobacillus*, *Clostridia*, and *Ruminococcaceae,* resulting in decreased production of SCFAs, including acetic acid, butyric acid, and propionic acid. Many animal studies and meta-analyses have reported that SCFAs can help mitigate metabolic diseases such as obesity, diabetes, and cardiovascular disease. SCFA levels reduced by HFD were partially restored by kimchi intake.

In addition to gut bacteria and SCFAs, kimchi intake partially restored HFD-induced alterations in blood LPCs, intestinal lipids, amino acids, and hepatic lipid metabolites (Figure 2), all of which are associated with abnormal lipid and energy metabolism (Figure 3). This restoration is supported by previous serum and liver metabolomic studies conducted in HFD-induced obese mice [9] and overweight/obese men [9]. Similar results were observed for green tea intake in HFD-induced obese mice [32]. Interestingly, despite the unclear role of urinary tartaric acid in the relationship between obesity and its physiological impact [33], its significant increase (40.5-fold) in the HFD group compared to the ND group suggests its potential as a biomarker of HFD-induced obesity. However, in contrast to our findings, a negative correlation has been found between total and LDL cholesterol levels and urinary tartaric acid levels in elderly men and women with type 2 diabetes mellitus or at least three cardiovascular risk factors [34]. In our study, the elevated LDL level in the KHD group compared to the HFD group may have been due to the high salt content [35] and certain compounds in kimchi that could have contributed to the higher LDL levels, despite the cholesterol-lowering effects of NDCs in kimchi. Moreover, urinary sorbitol and sulfuric acid were increased by kimchi intake, but not by the HFD, and could be used as biomarkers for kimchi consumption. Sorbitol, whose content in Korean commercial kimchi is substantially higher than that in commercial kimchi from China and the United States, is produced as a metabolite of sugar-based microorganisms [36], whereas sulfuric acid, which contributes to the unique flavor profile of kimchi, is generally produced from ingredients during kimchi fermentation.

Notably, we report for the first time the effects of kimchi intake on bile acids and steroid hormones in HFD-induced obese mice. Many clinical studies have revealed that bile acids and steroid hormones are closely associated with the regulation of obesity [37,38]. In particular, obesity has been consistently linked to alterations in sex steroid hormone levels in men, with a significant decrease in testosterone levels and an increase in estrone and estradiol [39,40]. In women, obesity is also linked to significant hormonal changes, specifically affecting estrogen levels, which can lead to an increased risk of metabolic and cardiovascular diseases due to enhanced aromatization in the adipose tissue [41]. Moreover, obesity in youth is associated with enhanced activities of all adrenocortical steroidogenic pathways, as reflected by increased excretion of mineralocorticoid derivatives, tetrahydro-derivatives of cortisol/cortisone, and androgen, whereas urine excretion of estrogens is independent of body mass and correlated with the age, sex, and puberty stage [42].

Moreover, non-12-OH bile acids, including HCA, HDCA, glycohyodeoxycholic acid (GHDCA), ursodeoxycholic acid (UDCA), chenodeoxycholic acid (CDCA), and GUDCA, significantly decreased in unhealthy subjects with high BMIs and are associated with altered gut microbiota [43]. Similar results were observed in the present study, although some findings were contradictory. Interestingly, kimchi intake partially reversed the HFD-induced alterations in bile acids and steroid hormones (Figure 4). Previous studies on factors affecting bile acids and steroid hormones have indirectly suggested that changes in the gut microbiota due to kimchi intake, along with kimchi-intake-induced endogenous metabolites such as SCFAs and kimchi components, including NDCs, minerals, and specific phytochemicals, might contribute to these observed changes [44]. Correlation data between gut microbiota and metabolites, bile acids, and steroid hormones showed that *Muribaculaceae* and *Lactobacillus* released by the HFD were positively correlated with steroid hormones and metabolites, while a negative correlation was observed for the *Eubacterium_xylanophilum_group* and *Ruminococcus* increased by the HFD (Figure 5). The correlation between the HFD and these bacteria has been previously demonstrated, showing that oral administration of *Lactobacillus plantarum* Y44 restored HFD-altered gut microbiota, along with obesity-associated parameters [45]. Although the data acquired in our study were insufficient to establish a definitive correlation with kimchi intake, they highlight the benefits of kimchi in the fight against HFD-induced obesity.

## 5. Conclusions

In conclusion, our study demonstrates the beneficial effects of a kimchi intake on HFD-induced mild obesity, notably demonstrating the partial amelioration of these effects and highlighting the significant influence of kimchi on gut microbiota, steroid hormones, bile acids, and metabolites. Based on these findings, we propose metabolomic and steroid hormone pathways associated with a HFD and kimchi intake, along with a correlation between gut microbiota and steroid hormones, bile acids, and metabolites. Although this study has several limitations, including the lack of a deeper understanding of the anti-obesity mechanisms of kimchi and the role of steroid hormones, our findings indicate that a kimchi intake could serve as a potential dietary intervention for the management of obesity and related diseases.

## Figures and Tables

**Table 1:** Biological characteristics of rat fed a normal diet, high-fat diet, and high-fat-kimchi diet.

	ND	HFD	KHD
weight gain (g)	199.55 ± 19.22 ^a^	237.57 ± 19.20 ^b^	208.71 ± 20.49 ^ab^
food intake (g/ day)	46.8 ± 0.89 ^b^	30.21 ± 2.29 ^a^	30.6 ± 1.556 ^a^
spleen (g)	0.72 ± 0.08 ^a^	0.7 ± 0.11 ^a^	0.6 ± 0.11 ^a^
liver (g)	11.85 ± 0.85 ^a^	11.08 ± 1.25 ^a^	11.30 ± 1.38 ^a^
kidney (g)	1.23 ± 0.39 ^a^	1.23 ± 0.09 ^a^	1.20 ± 0.14 ^a^
epididymal adipose tissue (g)	5.63 ± 1.08 ^a^	9.30 ± 2.95 ^b^	9.27 ± 1.52 ^b^
AST (U/L)	57.22 ± 7.47 ^a^	60.29 ± 15.59 ^a^	67.57 ± 10.67 ^a^
ALT (U/L)	38.33 ± 4.18 ^b^	28.28 ± 4.60 ^a^	31.00 ± 7.52 ^a^
TCHO (mg/dL)	114.44 ± 19.37 ^a^	111.57± 12.06 ^a^	112.85± 12.26 ^a^
TG (mg/dL)	45.11 ± 12.02 ^b^	36.14 ± 11.68 ^a^	35.71 ± 10.89 ^ab^
HDL (mg/dL)	45.88 ± 8.96 ^a^	49.43 ± 5.06 ^a^	59.14 ± 33.28 ^a^
LDH (U/L)	97.67± 35.01 ^a^	105.71 ± 36.76 ^a^	133.57 ± 77.58 ^a^

ND, normal diet; HFD, high-fat diet; KHD, high-fat diet with 5% kimchi; AST, alanine aminotransferase; ALT, aspartate aminotransferase; TCHO, total cholesterol, TG, triglyceride; HDL, high-density lipoprotein, LDL, low-density lipoprotein. Values are expressed as mean ± SD (*n* = 10) and different letters in the same column indicate significant differences in Duncan’s test at *p* < 0.05.

## Data Availability

The data presented in this study are available upon reasonable request from the corresponding author.

## Supplementary Materials

The following supporting information can be downloaded at: https://www.mdpi.com/article/10.3390/nu16183095/s1, Figure S1: RT-PCR for the assessment of inflammation and proinflammatory cytokine expression. Expression of RNA-encoding inflammation markers and proinflammatory cytokines such as IL-12, IFNg, IL-1b, IL-6, and TNF-*α*; Figure S2: Comparison of relative abundance of gut microbiota after eight weeks of kimchi intervention. (A) Bar charts summarizing overall microbial composition in feces of rats fed the ND, HFD and KHD at genus levels with the average relative abundance; Figure S3: The qualification of the PLS-DA models was evaluated by cross-validated with a permutation test; Table S1: UPLC-Q-TOF MS-MRM conditions for steroid hormone analysis; Table S2: UPLC-Q-TOF MS-MRM conditions for bile acid analysis; Table S3: The forward and reverse primer sequences used for real-time PCR; Table S4. Oral glucose tolerance test results of rat fed a normal diet, high-fat diet, and high-fat-kimchi diet; Table S5: The VIP scores and fold changes of metabolites analyzed by UPLC-Q-TOF MS; Table S6: The VIP scores and fold changes of metabolites analyzed using GC–MS.
