# Supplementary material for: Effects of Kimchi Intake on the Gut Microbiota and Metabolite Profiles of High-Fat-Induced Obese Rats
Source: Nutrients. 2024 Sep 13;16(18):3095. doi: 10.3390/nu16183095 (PMC11435375; doi:10.3390/nu16183095)
Supplement: Supplementary file 1 [file nutrients-16-03095-s001.zip › nutrients-3184343-supplementary.pdf]

Table S1. UPLC-Q-TOF MS-MRM conditions for steroid hormone analysis

NO	Compounds	MRM	Collision energy (eV)
1	Androsterone	291.2 > 273.2	10
2	epiandrosterone	273 > 97	30
3	allodihydrotestosterone	291.3 > 159	20
4	testosterone	289.2 > 271.2	30
5	17a-methyltestosterone	303 > 97	20
6	2-methoxy-3-OH-estradiol	303.3 > 137.2	20
7	cortexone	331.3 > 97.1	20
8	estiol-16-acetate	331.3 > 253.1	20
9	2,3-dimethoxyestradiol	317.3 > 302.2	10
10	21-deoxycortisol	347 > 293	20
11	21-deoxycortisol	347.2 > 269.1	20
12	21-deoxycortisol	347.2 > 311.1	10
13	estriol-16-hemisuccinate	389.3 > 253.1	20
14	estradiol-3,17a-diacetate	297.3 > 255.1	20
15	2,3-dimethoxyestrone	315.3 > 201	20
16	progesterone	315 > 97	30
17	estradiol-17-hemisuccinate	373.3 > 255.1	20
18	cortisol	363.4 > 121.1	20
19	16,17-epiestriol triacetate	415.3 > 295	10
20	estradiol-3-acetate	315.3 > 107.1	30
21	cortisone	361 > 105	30
22	pregnenolone	317.3 > 281.1	35
23	progesterone	315.3 > 97.1	20
24	3-methoxy-2-OH-estrone	301.3 > 137	20
25	epitestosterone	289 > 97	30
26	androsterone	273 > 255	20
27	androstenedione	287.2 > 269.1	10
28	2-hydroxyestriol	287.3 > 269	10
29	3-methoxy estriol	285.3 > 266.9	10/20
30	aldosterone	361 > 343	10
31	9,11-dehydro E1	271.3 > 159.3	20
32	2-methoxy-3-OH-estrone	301.3 > 189.2	20
33	estriol	271> 133	20
34	estrone	271.2> 253.2	10
35	DHEA	289.3>197	20
36	11-hydroxyestradiol	289.3> 253.1	20
37	Estriol-3-sulfate	367.2> 287.3	20
38	Estriol-3-hemisuccianate	255.3> 159.1	20
39	Estrone-3-acetate	313.3> 253.1	20
40	Estrone-3-hemisuccianate	371.2> 177.2	20
41	6-hydroxyl-estradiol	271.2> 156.9	20
42	pregnanediol	285.3> 81.1	20
43	methoxyprogesterone	345.2>123.0	20
44	ethisteron	360.0> 270.0	20
45	11b-OH-etiocholanolone	385.1> 448.0	20

46	5a-androtane-3a,17b diol	291.24> 255.22	20
47	4a-androtane-3,17 diol	301.26> 283.25	20

Table S2. UPLC-Q-TOF MS-MRM conditions for bile acid analysis

NO	Compounds	MRM	Collision energy (eV)
1	Taurocholic	462.2> 337.2	25
2	Dehydrocholic acid	385.2> 367.2	15
3	Cholic acid	355.2> 239.1	25
4	deoxycholic	357..2> 215.1	25
5	Hyocholic acid	355.2> 157.2	25
6	Chenodeoxycholic	357.2> 161.1	25
7	Glycochenodeoxycholic acid	414.2> 339.2	18
8	Hyodeoxycholic acid	357.2> 161.1	25
9	Taurohyodeoxycholic acid	464.2> 339.2	25
10	Glycoursodeoxycholic acid	414.2> 339.2	22
11	Tauroursodeoxycholate	464.2> 339.2	25
12	Tauro-α-muricholic Acid	514.2	6
13	Tauro-β-muricholic Acid	514.2	6
14	Tauro-ω-muricholic Acid	514.2	6

Table S3. The forward and reverse primer sequences used for Real-Time PCR

	Forward primer	Reverse primer
Il-12	5′-ATGATGACCCTGTGCCTTGG-3′	5′-TGCTGCATTTATGGCCTGGA-3′
Il-1b	5′-TCCTCTGTGACTCGTGGGAT-3′	5′-TCAGACAGCACGAGGCATTT-3′
Il-6	5′-TCCTACCCCAACTTCCAATGCTC-3′	5′-TTGGATGGTCTTGGTCCTTAGCC3′
IFN-γ	5′-CGAGGTGAACAACCCACAGA -3′	5′-CGACTCCTTTTCCGCTTCCT-3′
TNF-α	5′-CGTCAGCCGATTTGCCATTT-3′	5′-TCCCTCAGGGGTGTCCTTAG -3′
Actb	5′-AGATGACCCAGATCATGTTTGAGA-3′	5′-ACCAGAGGCATACAGGGACA -3′

Table S4. Oral glucose tolerance test results of rat fed a normal diet and high-fat diet and high-fat-kimchi diet

	Blood glucose (mg/dL)		
Time(min)	ND	HFD	KHD
0	98.00 ± 10.77[a]	104.14 ± 7.80[a]	114.43 ± 7.46[b]
30	134.33 ± 19.14[a]	164.86 ± 25.90[b]	150.43 ± 18.49[a]
60	154.00 ± 24.75[a]	176.00 ± 15.73[ab]	165.71 ± 30.24[ab]
90	136.78 ± 21.53[a]	171.43 ± 15.67[ab]	153.71 ± 18.94[ab]
120	125.22 ± 16.08[a]	166.29 ± 20.30[b]	144.71 ± 18.85[a]

ND, normal diet; HFD, high-fat diet; KHD, high-fat diet with 5% kimchi.
Values were expressed an mean ± SD (n=7) and different letters in the same column indicated significant differences Duncan's test at $p < 0.05$.

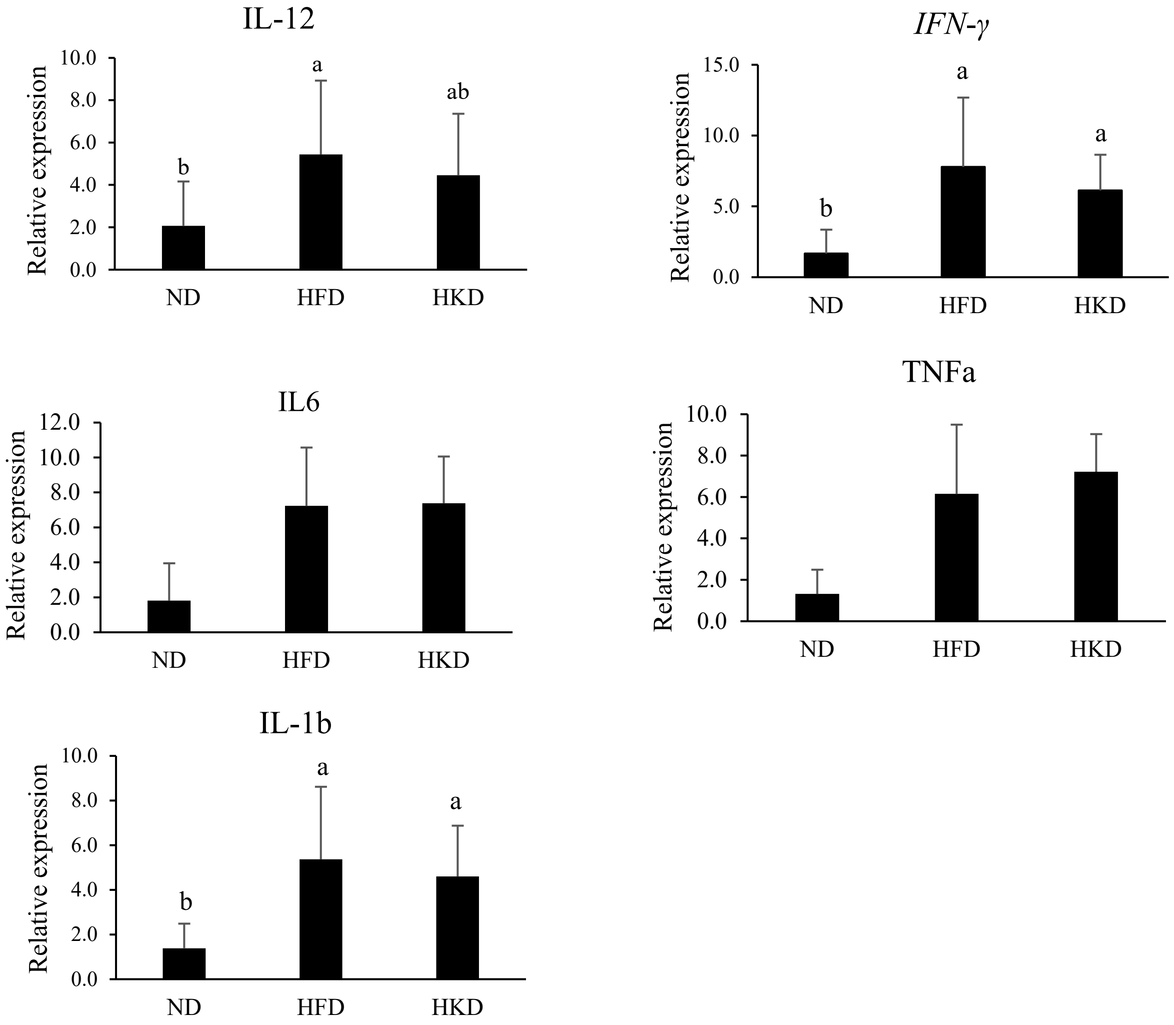

Figure S1. RT-PCR for the assessment of inflammation and proinflammatory cytokine expression. Expression of RNA encoding inflammation markers and proinflammatory cytokines such as IL-12, *IFN*-*γ*, IL-1b, IL-6, and TNF-*α*.

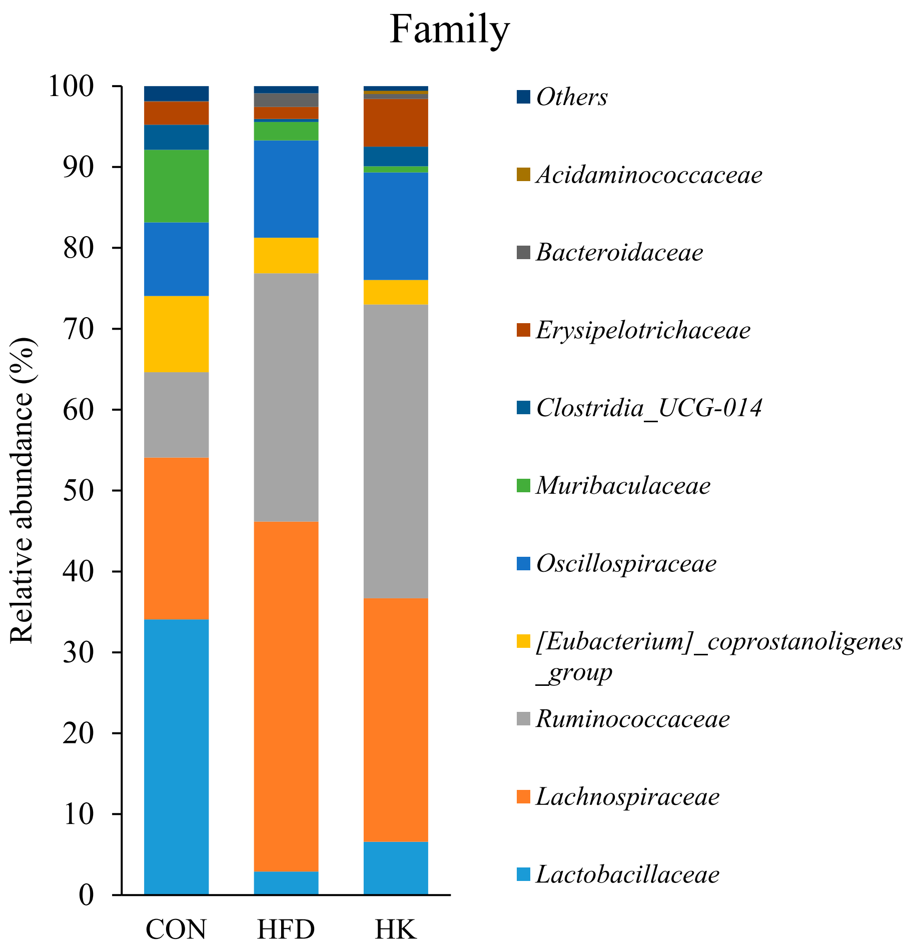

Figure S2. Comparison of relative abundance of gut microbiota after eight weeks of kimchi intervention. (A) Bar charts summarizing overall microbial composition in feces of rats fed ND, HFD and KHD diet at genus levels with the average relative abundance.

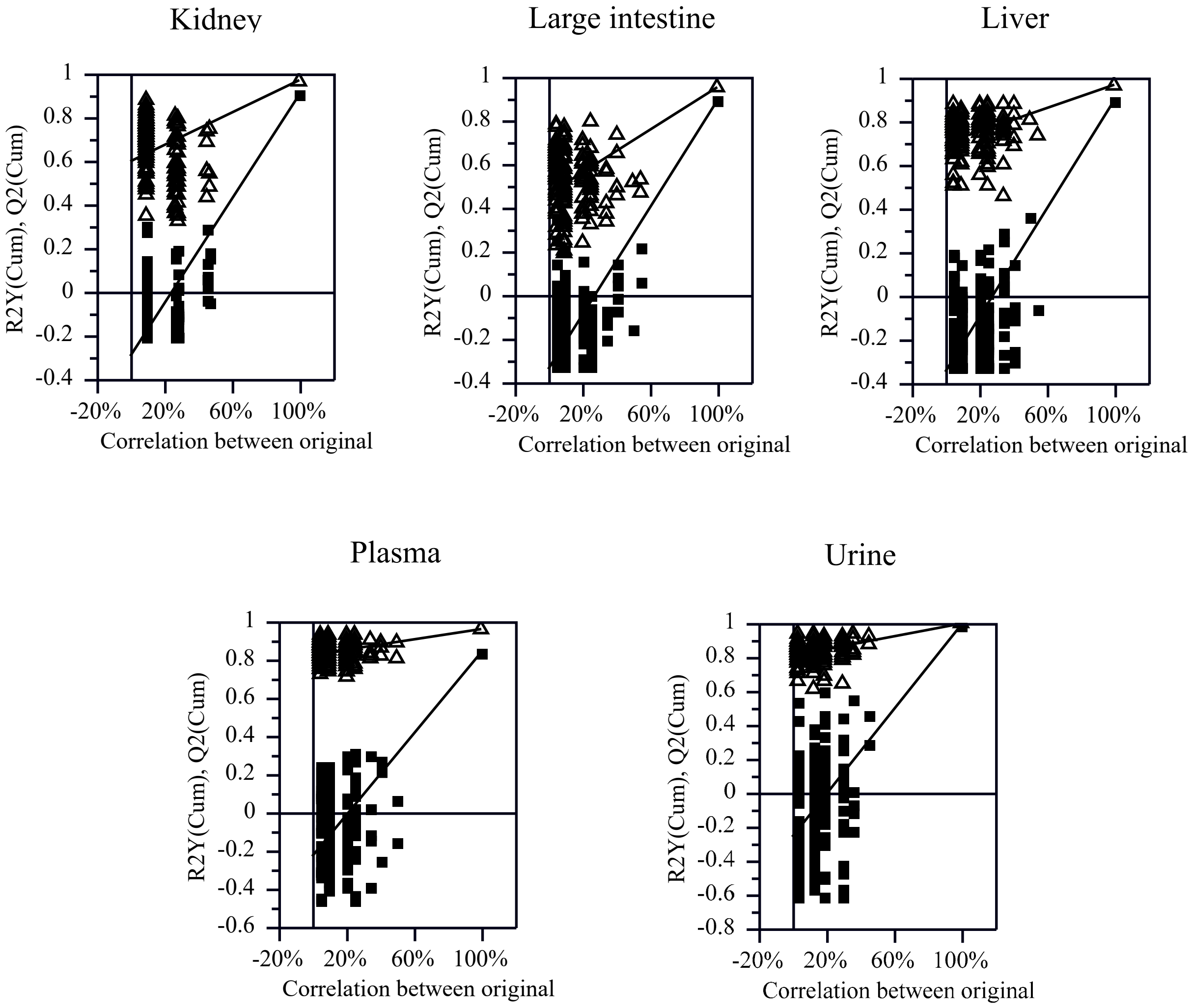

Figure S3. The qualification of the PLS-DA models was evaluated by cross-validated with permutation test

Table S5. The VIP scores and fold changes of metabolites analyzed by UPLC-Q-TOF MS

	Compound	*p*-value	VIP	Fold change (vs. ND)	
				HFD	KHD
**Plasma**	valine	2.11E-02	1.319	-1.175	-1.141
	phenylalanine	1.15E-02	1.425	-1.115	-1.313
	tryptophan	7.32E-02	1.148	-1.071	-1.167
	creatine	1.12E-04	1.86	-1.601	-1.589
	acetylcarnitine	4.10E-03	1.554	-1.108	-1.395
	palmitoylcarnitine	8.50E-02	1.104	2.120	1.541
	linolenic acid	1.52E-07	2.188	-2.037	-2.642
	LPE(C18:2)	4.43E-03	1.545	-1.458	-1.581
	LPC(C14:0)	1.79E-02	1.352	-2.304	-2.309
	LPC(C15:0)	7.85E-11	2.406	-4.399	-6.082
	LPC(C16:0)	1.62E-08	2.278	-1.503	-1.453
	LPC(C16:0) *	9.10E-08	2.223	-1.279	-1.221
	LPC(C16:1)	2.14E-08	2.271	-4.861	-3.632
	LPC(C17:0)	3.97E-11	1.266	-2.825	-3.327
	LPC(C17:1)	9.75E-06	2.009	2.452	2.557
	LPC(C18:0)	2.26E-03	1.612	1.376	1.288
	LPC(C18:0) *	1.04E-06	2.108	1.346	1.346
	LPC(C18:2) *	1.21E-08	2.289	-1.684	-1.485
	LPC(C20:1)	1.01E-04	1.867	-5.226	-3.455
	LPC(C20:2)	1.27E-06	2.113	-3.588	-2.431
	LPC(C20:3)	1.38E-02	1.339	-2.325	1.082
	LPC(C20:4) *	6.58E-02	1.108	-1.179	-1.051
**Urine**	pipecolic acid	9.88E-04	0.914	-1.287	-1.189
	pantothenic acid	3.84E-26	1.367	-6.968	-6.709
	inositol	3.14E-07	1.485	1.247	1.079
	ribosylimidazoleacetic acid	2.58E-07	1.153	-1.592	-1.413
	uric acid	3.54E-02	1.113	1.049	-1.042
	deoxycytidine	3.89E-02	0.703	2.451	1.899
	kynurenic acid	6.33E-02	0.81	-1.091	-1.250
	succinyladenosine	3.61E-03	1.082	1.275	1.083
	creatinine	4.59E-03	1.311	1.011	-1.125
	creatine	5.79E-02	0.988	1.013	-1.493
	carnitine	1.19E-03	1.392	1.462	-1.103
	m-methylglutaryl carnitine	3.00E-07	1.163	-3.159	-4.088
	acetylcarnitine	1.50E-02	0.863	1.504	1.233
	isovalerylcarnitine	4.40E-07	1.172	4.023	3.149

	Compound	*p*-value	VIP	Fold change (vs. ND)	
				HFD	KHD
**Liver**	phenylalanine	5.04E-03	1.427	-1.057	-1.155
	tryptophan	1.06E-02	1.442	-1.041	-1.210
	oxidized glutathion	1.22E-04	1.566	-1.574	-1.665
	adenosine	1.59E-03	1.387	1.331	1.347
	methylthioadenosine	8.27E-06	1.729	-1.350	-1.588
	glycerophosphocholine	7.82E-03	1.195	-1.273	-1.388
	NAD	6.97E-04	2.082	1.094	-1.254
	AMP	4.24E-04	1.518	-1.327	-1.722
	butyrylcarnitine	2.23E-02	1.114	-1.373	-1.535
	palmitoylcarnitine	5.50E-04	2.081	1.601	1.440
	LPE(C18:2)	4.68E-08	1.913	-3.480	-1.635
	LPC(C16:0) *	4.68E-08	1.848	-1.731	-1.883
	LPC(C18:1)	9.93E-07	1.812	-2.448	-2.458
**Intestinal content**	phenylalanine	3.72E-02	0.749	1.207	1.003
	tryptophan	3.12E-03	0.811	2.452	1.898
	stearoylcarnitine	3.14E-03	0.841	2.626	2.550
**Kidney**	phenylalanine	6.24E-03	1.249	-1.296	-1.246
	tryptophan	2.75E-05	2.102	1.253	-1.790
	ergothioneine	1.82E-13	1.629	-6.066	-6.256
	riboflavin	8.28E-02	1.201	-1.127	1.104
	phantothenic acid	3.84E-09	1.567	-1.577	-1.419
	pyridoxic acid	3.00E-04	1.21	-1.242	-1.279
	glycerophosphocholine	9.46E-03	1.491	1.002	-1.098
	citicoline	3.37E-02	1.036	1.413	1.195
	AMP	2.68E-02	0.882	1.116	1.098
	creatine	1.88E-03	1.826	1.336	-1.126
	carnitine	7.26E-03	1.138	-1.104	-1.182
	acetylcarnitine	1.15E-02	1.539	1.045	-1.084
	isovalerylcarnitine	5.54E-04	1.248	-1.551	-1.930
	myristoylcarnitine	6.55E-06	1.468	1.850	1.551
	palimitoylcarnitine	2.08E-07	1.497	1.669	1.521
	vaccenylcarnitine	3.07E-10	1.629	3.138	2.631
	stearoylcarnitine	2.27E-05	1.341	2.288	2.048
	LPE(C18:1)	1.33E-03	1.114	1.528	1.554
	LPE(C18:2)	5.41E-03	1.055	-1.355	-1.251
	LPE(C20:4)	5.66E-02	1.248	-1.042	1.143
	LPC(C16:0)	2.83E-08	1.507	-1.262	-1.219
	LPC(C16:0) *	4.62E-06	1.429	-1.402	-1.276
	LPC(C16:1)	1.60E-07	1.455	-	-
	LPC(C17:0)	3.78E-07	1.443	-2.470	-2.134
	LPC(C18:0)	1.32E-07	1.644	1.237	1.363
	LPC(C18:0) *	9.54E-08	1.632	1.112	1.013
	LPC(C18:1)	5.15E-05	1.338	1.687	1.894
	LPC(C18:2)	7.36E-08	1.564	-1.549	-1.447

Table S6. The VIP score and fold change of metabolites analyzed using GC–MS

	Compound	*p*-value	VIP	Fold change (vs. ND)	
				HFD	KHD
**Plasma**	alanine	7.02E-02	0.924	-1.398	-1.538
	valine	7.31E-05	1.724	-1.280	-1.045
	isoleucine	5.37E-02	0.932	-1.181	-1.482
	phenylalanine	9.05E-10	1.69	-1.209	-1.602
	tyrosine $(TMS)_3$	1.90E-07	1.581	-1.383	-1.618
	glutamic acid	4.20E-11	1.833	-1.742	-1.984
	proline	5.96E-06	1.542	-1.224	-1.429
	glycerol	1.09E-03	1.206	-1.029	-1.337
	phosphoric acid	2.46E-07	1.591	-1.397	-1.480
	glutaric acid	1.75E-03	1.171	-1.054	-1.639
	3-hydroxybutyric acid	1.83E-02	1.346	1.475	-1.045
	creatinine	4.53E-03	1.223	-1.804	-2.244
	inositol	1.17E-05	1.852	-1.747	-1.858
	glucose MEOX $(TMS)_5$	2.63E-08	1.631	-1.128	-1.302
	glucose MEOX $(TMS)_5$	4.75E-12	1.762	-1.175	-1.368
	plamitic acid	3.44E-02	1.709	1.128	-1.208
	linoleic acid	2.55E-04	1.169	-1.468	-2.145
	octadecenoic acid	2.53E-03	2.54	1.808	1.195
	stearic acid	2.56E-02	1.324	1.039	-1.345
	unknown sterol	5.04E-02	0.695	-1.146	-1.356
**Urine**	oxalic acid	2.03E-03	0.928	-1.469	-2.391
	acetic acid	6.02E-02	0.807	1.565	1.445
	sulfuric acid	3.13E-03	0.807	1.056	1.901
	succinic acid	1.50E-03	0.962	-1.648	-2.105
	citric acid	5.80E-02	0.885	1.098	-1.426
	gluconic acid	6.58E-03	0.963	-1.147	-1.439
	pantothenic acid	5.40E-19	1.549	–	–
	erythritol	6.49E-02	0.625	1.131	1.281
	uric acid	6.85E-02	0.95	1.368	-1.065
	creatinine	1.37E-03	1.299	1.634	1.164
	tartaric acid	4.52E-13	1.464	40.501	38.192
	xylitol	4.01E-03	0.927	-1.379	-1.606
	arabitol	9.58E-13	1.451	-2.787	-2.948
	ribitol	2.22E-08	1.313	-2.094	-2.562
	glucose MEOX $(TMS)_5$	1.03E-02	0.931	-1.123	-1.376
	sorbitol	4.41E-11	1.363	-2.282	4.042
	sucrose	2.79E-04	1.241	1.992	1.508
	lactose	3.84E-02	0.909	-1.064	-1.288
	palmitic acid	3.24E-03	0.992	-1.118	-1.295
	stearic acid	1.46E-03	1.026	-1.134	-1.316

	Compound	*p*-value	VIP	Fold change (vs. ND)	
				HFD	KHD
**Liver**	valine	1.83E-02	1.346	1.136	-1.048
	glycine	1.09E-03	1.206	1.470	1.335
	theronine	5.37E-02	0.932	1.295	1.122
	lysine	4.53E-03	1.223	1.350	1.134
	phosphoric acid	7.31E-05	1.724	-1.097	-1.238
	threonic acid	1.75E-03	1.171	1.303	1.213
	3-hydroxybutyric acid	7.02E-02	0.924	1.453	1.131
	glycerol	2.46E-07	1.591	2.877	2.197
	inositol	4.20E-11	1.833	-1.330	-1.378
	myristic acid	5.96E-06	1.542	1.718	1.368
	palmitic acid	9.05E-10	1.69	2.304	2.006
	linoleic acid	2.63E-08	1.631	2.304	2.006
	octadecenoic acid	4.75E-12	1.762	6.162	5.103
	oleic acid	1.90E-07	1.581	1.971	1.686
	stearic acid	1.43E-07	1.581	1.551	1.430
	arachidonic acid	3.12E-14	1.816	7.158	5.232
**Intestinal content**	hydroxyamine	9.51E-03	1.326	1.534	-1.838
	alanine	7.02E-02	1.011	2.133	1.570
	valine	2.62E-02	1.136	2.775	1.930
	isoleucine	1.44E-02	1.173	3.091	2.328
	serine	9.08E-03	1.236	3.412	2.405
	threonine	1.21E-02	1.21	3.285	2.278
	glycine	1.11E-02	1.223	3.061	1.970
	glutamic acid	4.82E-02	1.039	2.166	1.670
	aspartic acid	7.09E-03	1.243	3.267	2.456
	oxalic acid	1.68E-02	1.162	3.748	1.758
	succinic acid	1.39E-02	1.197	1.441	-1.283
	phosphoric acid	6.45E-03	1.234	3.856	2.774
	ethanolamine	2.37E-02	1.115	1.881	1.474
	glycerol	7.98E-03	1.197	2.269	1.171
	glucose MEOX $(TMS)_5$	2.95E-03	1.468	-1.957	-2.038
	myristic acid	2.60E-02	1.077	2.521	1.814
	pentadecanoic acid	5.03E-02	1.259	-1.113	-1.532
	palmitic acid	4.84E-06	1.721	2.488	1.910
	octadecenoic acid	6.81E-04	1.413	6.984	4.074
	oleic acid	5.58E-03	1.247	2.427	1.702
	stearic acid	5.04E-03	1.257	2.074	1.620
**Kidney**	3-hydroxybutyric acid	1.73E-02	1.558	-1.443	-1.305
	dihydroxybutanoic acid	1.64E-03	1.894	-1.677	-2.101
	creatinine	7.14E-02	1.279	1.471	-0.788
	octadecenoic acid	1.12E-03	1.961	-1.785	-1.621
	serine	1.27E-04	2.134	-1.277	-1.342
	threonine	1.17E-02	1.652	-1.182	-1.237
	threonic acid	6.45E-02	1.347	-1.182	-1.237
	glycerophosphate	3.41E-02	1.452	-1.045	-1.228
